# Differential of Frequency and Duration Mismatch Negativity and Theta Power Deficits in First-Episode and Chronic Schizophrenia

**DOI:** 10.3389/fnbeh.2019.00037

**Published:** 2019-03-06

**Authors:** Yan-Bing Xiong, Qi-Jing Bo, Chang-Ming Wang, Qing Tian, Yi Liu, Chuan-Yue Wang

**Affiliations:** ^1^Department of Psychiatry, Beijing Anding Hospital, Capital Medical University, Beijing, China; ^2^Beijing Key Laboratory of Mental Disorders, Beijing, China; ^3^Beijing Institute for Brain Disorders Center of Schizophrenia, Beijing, China; ^4^The National Clinical Research Center for Mental Disorders, Beijing, China

**Keywords:** schizophrenia, mismatch negativity, first-episode schizophrenia, chronic schizophrenia, time-frequency analysis

## Abstract

**Background:** Due to its impairment in patients with schizophrenia, mismatch negativity (MMN) generation has been identified as a potential biomarker for identifying primary impairments in auditory sensory processing. This study aimed to investigate the dysfunctional differences in different MMN deviants and evoked theta power in patients with first-episode schizophrenia (FES) and chronic schizophrenia (CS).

**Methods:** We measured frequency and duration MMN from 40 FES, 40 CS, and 40 healthy controls (HC). Evoked theta power was analyzed by event-related spectral perturbation (ERSP) approaches.

**Results:** Deficits in duration MMN were observed in both FES (*p* = 0.048, Bonferroni-adjusted) and CS (*p* < 0.001, Bonferroni-adjusted). However, deficits in frequency MMN were restricted to the CS (*p* < 0.001, Bonferroni-adjusted). Evoked theta power deficits were observed in both patient groups when compared with the HC (*p _FES_* = 0.001, *p _CS_* < 0.001, Bonferroni-adjusted), yet no significant differences were found between FES and CS. Frequency MMN was correlated with the MATRICS consensus cognitive battery (MCCB) combined score (*r* = -0.327, *p* < 0.05) and MCCB verbal learning (*r* = -0.328, *p* < 0.05) in FES. Evoked theta power was correlated with MCCB working memory in both FES (*r* = 0.347, *p* < 0.05) and CS (*r* = 0.408, *p* < 0.01).

**Conclusion:** These findings suggest that duration MMN and evoked theta power deficits may be more sensitive for detection of schizophrenia during its early stages. Moreover, frequency MMN and theta power could potentially linked to poor cognitive functioning in schizophrenic patients. The findings mentioned above indicated that the neural mechanisms of the three indexes may vary between people.

## Introduction

One of the central features of schizophrenia is cognitive impairment (Elvevåg and Goldberg, 2000; Weickert et al., 2000), which may include both higher-order functions, such as working memory, and essential sensory functions like auditory function (Javitt and Freedman, 2015; Javitt and Sweet, 2015). Mismatch negativity (MMN) is a negative component of the auditory event-related potential (ERP), which may be indicative of the neural mechanisms of cognitive dysfunction that occur in patients with schizophrenia (Näätänen et al., 2014; Hay et al., 2015, Javitt and Sweet, 2015). In addition, MMN is a key component of auditory and visual change detection in the environment (Randeniya et al., 2018). MMN deficits have been identified in patients with first-episode schizophrenia (FES; Hermens et al., 2010; Mondragón-Maya et al., 2013), patients with chronic schizophrenia (CS; Salisbury et al., 2002; Magno et al., 2008), and ultra-high risk individuals (UHR; Higuchi et al., 2014; Perez et al., 2014). In return, MMN has been proposed as a biomarker for the early detection of patients with schizophrenia.

Auditory MMN is typically induced by a response to the auditory oddball paradigm in which repeating standards are interrupted by rare deviant stimuli, which could differ from the standards for multiple characteristics (Näätänen et al., 2001). MMN deficits have been widely investigated in relation to frequency and duration MMN deviants in patients with schizophrenia. However, the relative degree of these deficits remains to be elucidated (Avissar et al., 2017). Previous studies (Salisbury et al., 2002; Magno et al., 2008) found that frequency MMN deficits were common in CS, yet these deficits were not detected in FES. However, duration MMN deficits have been detected in both FES and CS. For this reason, frequency MMN may be an unreliable biomarker for diagnosing patients with schizophrenia during the early stages of this disease, especially when compared with duration MMN (Haigh et al., 2017).

Despite providing comprehensive information at a physiological level, ERP analyses of MMN offer limited regional details. In contrast to ERP analysis, neuro-oscillatory (event-related spectral perturbation, ERSP) approaches can provide information about underlying ERP disturbances at the circuit and molecular levels simultaneously (Javitt and Sweet, 2015). Previously, auditory MMN was shown to have primary evoked power within the theta frequency band (4–7 Hz) (Fuentemilla et al., 2008; Hsiao et al., 2009, Javitt, 2015), which is the band closely tied to the function of somatostatin (SST)-expressing (Womelsdorf et al., 2014) and multipolar bursting-type (Blatow et al., 2003) GABA interneurons. Some studies have already found that MMN-evoked theta power was impaired in patients with schizophrenia (Javitt et al., 2017). However, no studies have reported whether there is a difference in evoked theta power deficits between FES and CS.

In order to study the mechanism of MMN impairment in patients with schizophrenia, researchers associate it with clinical symptoms and cognition. When pertaining to the symptom domains in schizophrenia, relations to MMN amplitude deficits have not been detected at this time. Some reports have shown that negative symptoms may be associated with MMN deficits (Grzella et al., 2001; Salisbury et al., 2002), while other studies have found an association with positive symptoms (Thönnessen et al., 2008; Fisher et al., 2011). However, one meta-analysis failed to detect a relationship between MMN amplitude deficits and symptom domains (Umbricht and Krljes, 2005). Interestingly, antipsychotic drugs do not improve MMN amplitude (Umbricht et al., 1999; Oranje et al., 2017). D-serine, which functions as an endogenous ligand for the glycine modulatory site of the N-methyl-d-aspartate-type (NMDAR), was previously found to improve MMN amplitude in patients with schizophrenia (Kantrowitz et al., 2017). More importantly, many studies have also repeatedly demonstrated that NMDAR antagonists (ketamine or MK-801) can reduce MMN (Catts et al., 2016), suggesting that MMN generation is associated with NMDAR, but not dopamine receptors.

Recently, researchers have shifted their focus to the correlation between MMN measures and cognition impairment. Some studies reported significant correlations between duration MMN amplitude and cognitive functioning in the domains of verbal fluency (Higuchi et al., 2013), social cognition (Wynn et al., 2010), and executive functioning (Toyomaki et al., 2008). However, a recent study reported significant relationships between MMN amplitude with frequency deviants and cognitive function in the verbal learning domain, but there were no significant relationships with duration deviants (Lee et al., 2017b). Regarding MMN-evoked theta power, D-serine treatment can also significantly improve theta response (Kantrowitz et al., 2016). More specifically, the improvement in theta occurs during the preparation interval, which correlates explicitly with an increase in auditory cognitive abilities (Kantrowitz et al., 2017).

In general, MMN amplitude deficits have been observed in patients with schizophrenia. However, the basis for the difference between frequency and duration MMN deficits in patients with schizophrenia remains to be elucidated. In this study, we hypothesized that the neural mechanisms of duration and frequency MMN deficits are different and deficits occur during various stages of schizophrenia. Therefore, we collected frequency and duration MMN amplitude data from FES and CS to verify our hypothesis. Meanwhile, we used ERSP approaches to acquire the evoked power to the MMN standard stimulus. To the best of our knowledge, this is the first study to concurrently assess the electrophysiological indices of evoked power in both FES and CS. We hypothesized that the evoked power deficits in CS are worse than the FES, similar to the MMN amplitude. Lastly, we analyzed the relationship between MMN measures (amplitude and evoked theta power) and clinical symptoms, sociodemographic measures and cognition impairments to further explore the neural mechanism.

## Materials and Methods

### Participants

Eighty-five patients with schizophrenia were recruited from Beijing Anding Hospital, Capital Medical University. For inclusion in this study, the diagnoses were validated with the Structured Clinical Interview for DSM-IV (SCID). FES was classified as appearing within three years of entry into this study, while CS appeared more than five years before entry into this study. HC were recruited by advertisement and had no DSM-IV axis I disorders.

The age range was 18–45 years old, and all participants had IQs ≥ 70. The exclusion criteria for this study included hearing disorders, learning disabilities, neurological impairments, and histories of seizures, head injuries, electroconvulsive therapy, and drug abuse. The Ethics Committee of Beijing Anding Hospital reviewed and approved this study and all subjects provided informed consent prior to inclusion.

### Procedures

The auditory stimuli consisted of a sequence of binaural tones (825 trials) that were presented in random order with a stimulus onset asynchrony of 500–550 ms. Standard tones (675 trials, 82%) were 1000 Hz, 75 dB, and 50 ms in duration. Deviant tones included frequency and duration deviants. Frequency deviants (75 trials, 9%) were 1500 Hz, 75 dB, and 50 ms in duration. Duration deviants (75 trials, 9%) were 1000 Hz, 75 dB, and 100 ms in duration. At the beginning of the paradigm, the first 15 stimuli were used as the standard.

### Electroencephalogram (EEG) Data Acquisition and Processing

Electroencephalogram (EEG) data were collected from all of the subjects using the 128-channel electrode system (Electrical Geodesics, Inc., Eugene, OR, United States) with ground procedures and standard reference. The impedance of the signal was adjusted to ≥50 KΩ with a sampling rate of 1000 Hz. During the experiment, the subjects were seated comfortably in a light and sound-attenuated room to remove potentially interfering variables from the study. The test consisted of three sections with a 60 s break between each section.

**Figure 1:**
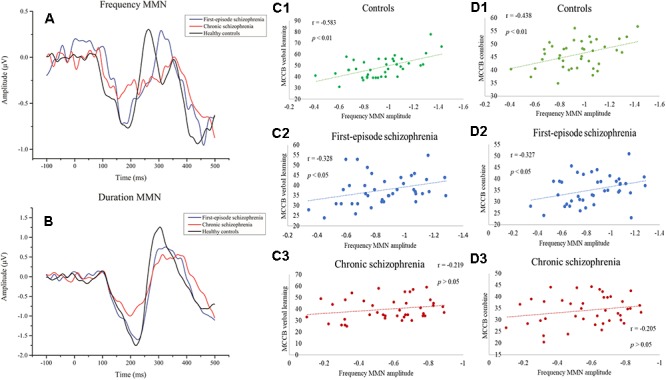
**(A)** Event-related potential (ERP) waveforms to frequency deviant stimuli. **(B)** ERP waveforms to duration deviant stimuli. **(C1–C3)** Correlations between frequency MMN and MCCB verbal learning showing a significant relationship with the healthy control group and first-episode schizophrenia group. **(D1–D3)** Correlations between frequency MMN and the mean MCCB combined score showing a significant association between the healthy control group and the first-episode schizophrenia group.

For the ERP analyses, the EEG data were analyzed and processed using EEGLAB 14.1.1b^1^, which is a neural electrophysiological analysis tool based on MATLAB (MathWorks, Natick, MA, United States). The EEG data were processed using a 0.1–40 Hz bandpass filter (finite impulse response filter). The 50 Hz power frequency noise was subject to notch processing. The reference electrode was changed to a global brain average reference. Artifacts due to eye movement were excluded by independent component analysis (ICA; Makeig et al., 1997). The EEG was segmented from 100 ms prior to initiation to 500 ms after the stimulus onset. MMN waveforms were collected by subtracting the standard from the deviant stimulus at the frontal midline (Fz) electrode (Figure 1A,B).

**Figure 2:**
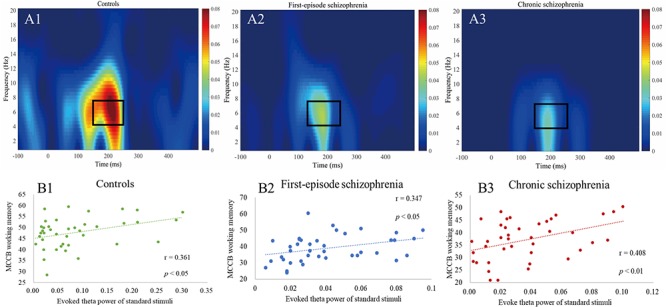
**(A1–A3)** Evoked power plots for standard stimuli. Box indicates the integration window for the theta band response (4–7 Hz, 150–250 ms). **(B1–B3)** Correlations between evoked theta power and MCCB working memory showing a significant relationship between all three groups.

For evoked (average) power analyses, ERP waves were transformed with the short-time Fourier transformation (STFT) method using MATLAB (MathWorks, Natick, MA, United States). Continuous wavelet transformation was carried out for the segmented EEG signal time. The EEG time range was from 100 ms before initiation to 500 ms after the stimulus onset, which was relative to the stimulus presentation time. The frequency range of the wavelet transformation was 1–20 Hz. Additionally, the temporal values of power corresponding to each frequency point were averaged across the trials, and thus EEG power time-frequency distribution was attained channel by channel. We extracted the maximum power values between 1–20 Hz and 100–250 ms of each subject for statistical analysis, and the range of the maximum value is within the theta frequency band of 4–7 Hz. We considered the theta frequency band to be the primary active frequency band of the nerve oscillation to the standard stimulus (Figure 2A1–A3).

### Clinical, Intelligence Quotient and Neuropsychological Assessment

The clinical symptoms of each patient were evaluated with the Positive and Negative Symptom Scale (PANSS, Chinese version), which was described previously (He and Zhang, 1997). The Chinese intelligence quotient (IQ) test tool is a revised short form of the Wechsler adult intelligence scale-revised, and the four included subsets for this evaluation were information, similarities, picture completion, and block design (Pang et al., 2011). The MATRICS consensus cognitive battery (MCCB, Chinese version) was used to evaluated cognitive deficits in patients with schizophrenia and healthy controls (Shi et al., 2015).

### Statistical Analysis

Data analyses were performed using SPSS 20.0 (IBM, Chicago, IL, United States). Continuous variables were checked using one-way analysis of variance (ANOVA) and classified variables using the Chi-square test. Analysis of covariance was used to adjust for confounding effects of cognition among groups. A two-factorial mixed ANOVA with deviant type as within-subject factor and the between-subject factor groups was carried out. Associations between MMN values and the scores from the MCCB tasks or the PANSS scale were analyzed using Pearson’s correlation analysis. The statistical test used a significance level of *p* < 0.05. *P*-values were corrected using Bonferroni adjustment (B-adjusted). Cohen’s effect size was used to analyze the differences between the means of each group. Measurement of the effect size was based on the Cohen coefficient (Cohen, 1988) with *d* < 0.2 as a negligible effect size, 0.2–0.5 as a small effect size, 0.5–0.8 as a medium effect size, and *d* > 0.8 as a large effect size.

## Results

### Demographics and Clinical Characteristics of Patients

Three FES and two CS were excluded from this study due to low-quality EEG data. In total, 40 FES, 40 CS, and 40 HC were enrolled in the study. The demographic characteristics and clinical data for the remaining participants are summarized in Table 1. There were no significant differences in age (*df* = 2,117, *F* = 2.742, *p* = 0.069), gender (χ^2^ = 1.364, *p* = 0.506), or education (*df* = 2, 117, *F* = 1.476, *p* = 0.233) among the three groups.

**Table 1:** Demographic and clinical characteristics of patients with first-episode schizophrenia, chronic schizophrenia, and healthy controls.

Variable	Group						Analysis			*post hoc* Tests *p*, Bonferroni-adjusted		
	FES (*N* = 40)		CS (*N* = 40)		HC (*N* = 40)		*df*	*F*	*p*	FES vs. CS	FES vs. HC	CS vs. HC
	Mean	*SD*	Mean	*SD*	Mean	*SD*						
Age (years)	25.63	6.21	28.50	5.34	26.33	5.60	2, 117	2.742	0.069	0.08	1.000	0.276
Education (years)	13.40	2.87	13.88	2.62	14.48	2.91	2, 117	1.476	0.233	1.000	0.267	1.000
IQ	101.48	12.403	105.58	9.732	114.1	13.345	2, 117	11.667	<0.001	0.381	<0.001	0.005
Age at illness onset (years)	24.48	6.27	21.35	4.84	–	–	1, 78	6.231	0.015	–	–	–
Duration of illness (months)	14.15	9.24	90.03	37.71	–	–	1, 78	152.8	<0.001	–	–	–
**Positive and Negative Syndrome Scale**
Positive scale score	20.98	5.55	21.60	3.95	–	–	1, 78	0.337	0.563	–	–	–
Negative scale score	20.35	5.90	19.65	4.80	–	–	1, 78	0.339	0.562	–	–	–
General psychopathology score	40.1	6.11	42.18	5.57	–	–	1,78	2.524	0.116	–	–	–
PANSS score	81.43	12.33	83.43	10.25	–	–	1,78	0.622	0.433	–	–	–
	N	%	N	%	N	%	df	χ2	*p*	FES vs. CS	FES vs. HC	CS vs. HC
Sex (Male, %)	28	70	23	57.5	25	62.5	2	1.364	0.506	0.245	0.478	0.648
**Antipsychotic treatment**
None	24	60	4	10	–	–	–	–	–	–	–	–
First-generation antipsychotic	0	0	0	0	–	–	–	–	–	–	–	–
Second-generation antipsychotic	16	40	36	90	–	–	–	–	–	–	–	–

^∗^*FES = First-episode Schizophrenia; CS = Chronic Schizophrenia; HC = Healthy Controls; IQ = intelligence quotient; – = Not Available*.

As expected, the HC had a higher IQ (*df* = 2, 117, *F* = 11.667, *p* < 0.001) and better MCCB task performance than FES and CS. The MCCB domain scores and statistical analyses are shown in Table 2. In analyzing covariance for IQ, the differences among groups remained significant for all the MCCB domains (*df* = 2, 117, *F*_speed of processing_ = 27.548, *p* < 0.001; *F*_attention/vigilance_ = 37.489, *p* < 0.001; *F*_working memory_ = 13.318, *p* < 0.001; *F*_verbal learning_ = 7.53, *p* = 0.001; *F*_visual learning_ = 3.69, *p* = 0.028; *F*_reasoning and problemsolving_ = 11.035, *p* < 0.001, *F*_social cognition_ = 3.321, *p* = 0.04; *F*_MCCB combine_ = 31.889, *p* < 0.001).

**Table 2:** MATRICS consensus cognitive battery (MCCB) domain-scores for first-episode schizophrenia, chronic schizophrenia, and healthy control groups.

Variable	Group						Analysis			*post hoc* Tests *p*, Bonferroni-adjusted		
	FES (*N* = 40)		CS (*N* = 40)		HC (*N* = 40)		*df*	*F*	*p*	FES vs. CS	FES vs. HC	CS vs. HC
	Mean	*SD*	Mean	*SD*	Mean	*SD*						
Speed of processing	35.88	7.65	34.88	8.09	48.18	6.13	2, 117	40.778	<0.001	1.000	<0.001	<0.001
Attention/Vigilance	29.58	10.64	24.7	12.23	46.4	7.44	2, 117	48.907	<0.001	0.109	<0.001	<0.001
Working memory	39.25	8.23	37.45	7.96	48.1	6.95	2, 117	21.736	<0.001	0.900	<0.001	<0.001
Verbal learning	37.78	7.28	39.93	9.49	48.4	9.17	2, 117	16.67	<0.001	0.814	<0.001	<0.001
Visual learning	41.83	11.32	39.2	11.68	48.5	11.5	2, 117	7.738	0.001	0.851	0.021	0.001
Reasoning and problem solving	33.28	10.57	33.53	8.59	45.6	9.87	2, 117	21.054	<0.001	1.000	<0.001	< 0.001
Social cognition	29.95	9.81	29.8	11.31	37.45	9.57	2, 117	7.223	0.001	1.000	0.004	0.004
MCCB combine	35.6	6.41	34.4	6.53	46.1	5.29	2, 117	44.524	<0.001	1.000	<0.001	< 0.001

^∗^*FES = First-episode Schizophrenia; CS = Chronic Schizophrenia; HC = Healthy Controls*.

### Mismatch Negativity to Different Deviants

Results showing the differences in frequency MMN, duration MMN, and standard ERSP between the three groups are shown in Table 3. More specifically, these findings describe the relative magnitude of MMN deficits between the groups. As predicted, there was a significant difference among the three groups in both frequency MMN (*df* = 2, 117, *F* = 30.968, *p* < 0.001) and duration MMN (*df* = 2, 117, *F* = 23.962, *p* < 0.001).

**Table 3:** Mismatch negativity amplitudes and event-related spectral perturbation indexes.

Variable	Group						Analysis			*post hoc* Tests *p*, Bonferroni-adjusted			Cohen’s d	
	FES (*N* = 40)		CS (*N* = 40)		HC (*N* = 40)		*df*	*F*	*p*	FES vs. CS	FES vs. HC	CS vs. HC	FES vs. HC	CS vs. HC
	Mean	*SD*	Mean	*SD*	Mean	*SD*						
Frequency MMN (μV)	-0.8557	0.2295	-0.57	0.2102	-0.94	0.221	2, 117	30.968	<0.001	<0.001	0.269	< 0.001	0.37	1.72
Duration MMN (μV)	-1.6366	0.4801	-0.9146	0.3035	-1.8893	0.5654	2, 117	47.802	<0.001	< 0.001	0.048	< 0.001	0.48	2.15
Standard ERSP	0.0411	0.0249	0.0366	0.0276	0.0846	0.0793	2, 117	10.98	<0.001	1.000	0.001	< 0.001	0.74	0.81

^∗^*FES = First-episode Schizophrenia; CS = Chronic Schizophrenia; HC = Healthy Controls; MMN = Mismatch Negativity*.

When compared to HC, there was no significant difference with FES in terms of frequency MMN (*p* = 0.269, Bonferroni-adjusted), and the Cohen’s effect size was small (*d* = 0.37). However, the difference was significant (*p* < 0.001, Bonferroni-adjusted) and the Cohen’s effect size was large (*d* = 1.72) when comparing CS with HC. Meanwhile, in terms of duration MMN, both FES (*p* = 0.048, Bonferroni-adjusted) and CS (*p* < 0.001, Bonferroni-adjusted) showed significant differences when compared with HC. The Cohen’s effect size for FES and CS were approximately medium (*d* = 0.48) and large (*d* = 2.15), respectively. When the two patient groups were compared, there was a significant main effect for both frequency MMN (*p_F_* < 0.001, Bonferroni-adjusted) and duration MMN (*p_d_* < 0.001, Bonferroni-adjusted).

The results of two-factorial mixed ANOVA with the deviant type (frequency and duration) as within-subject and between-subject factor groups (FES and CS) showed that the effects within subjects were significant (*F* = 167.023, *p* < 0.001), and the effects between subjects were also significant (*F* = 75.835, *p* < 0.001). The interaction effects with MMN type (frequency vs. Duration) ^∗^ Group were also significant (*F* = 25.112, *p* < 0.001).

The relationship between MMN amplitude and several variables, including sociodemographic variables, MCCB tests, and PANSS scale scores, are described in Tables 4, 5. As we expected, no significant correlations were found between MMN amplitude and sociodemographic variables or MMN amplitude and PANSS scale scores. In terms of the MCCB tests, both FES (*r* = -0.327, *p* = 0.039, Figure 1D2) and HC frequency MMN (*r* = -0.438, *p* = 0.005, Figure 1D1) were strongly correlated with overall cognitive functioning, as assessed by the combined MCCB scores. However, CS frequency MMN was not associated with overall cognitive functioning. Of the three groups, only the duration MMN (*r* = -0.341, *p* = 0.032) of the healthy control group was found to correlate with overall cognitive function. Among the individual MCCB items, the correlation between frequency MMN and verbal learning was detected in FES (*r* = -0.328, *p* = 0.039, Figure 1C2) and HC (*r* = -0.583, *p* < 0.001, Figure 1C1), yet it was not observed in CS. In contrast, HC showed weak correlations between duration MMN and speed of processing (*r* = -0.326, *p* = 0.04), as well as duration MMN and social cognition (*r* = -0.335, *p* = 0.034). However, when using the Bonferroni correction, only the correlation between frequency MMN and MCCB verbal learning in HC was statistically significant, suggesting that the respective correlations are only significant on a descriptive level.

**Table 4:** Correlation between mismatch negativity measures with age, age at illness onset, duration of illness, and PANSS scores.

Variable	FES (*n* = 40) (r)			CS (*n* = 40) (r)		
	Frequency MMN	Duration MMN	Standard ERSP	Frequency MMN	Duration MMN	Standard ERSP
Positive scale score	0.026	-0.036	-0.176	-0.149	0.051	-0.142
Negative scale score	0.167	0.140	0.002	0.198	0.179	-0.395^∗^
General psychopathology score	-0.074	-1.113	0.025	0.159	0.118	-0.144
PANSS score	0.055	-0.005	-0.053	0.122	0.168	-0.318^∗^
Age (years)	0.022	-0.057	0.228	0.09	-0.185	0.176
Age at illness onset (years)	-0.007	-0.062	0.256	0.202	-0.140	0.193
Duration of illness (months)	0.215	0.095	-0.236	-0.13	-0.138	0.002

^∗^*FES = First-episode Schizophrenia; CS = Chronic Schizophrenia; MMN = Mismatch Negativity; ERSP = event-related spectral perturbation; PANSS = Positive and Negative Symptom Scale; ^∗^p < 0.05*.

**Table 5:** Correlation between mismatch negativity measures and the MATRICS consensus cognitive battery domain.

Variable	FES (*n* = 40) (r)			CS (*n* = 40) (r)			HC (*n* = 40) (r)		
		
	Frequency MMN	Duration MMN	Standard ERSP	Frequency MMN	Duration MMN	Standard ERSP	Frequency MMN	Duration MMN	Standard ERSP
Speed of processing	-0.276	-1.115	0.162	-0.126	-0.064	0.202	-0.286	-0.326^∗^	0.158
Attention/Vigilance	-0.301	-0.066	0.191	-0.179	0.072	-0.257	-0.136	-0.036	0.091
Working memory	-0.146	0.01	0.347^∗^	-0.095	-0.091	0.408^∗∗^	-0.241	-0.13	0.361^∗^
Verbal learning	-0.328^∗^	-0.008	-0.193	-0.219	-0.084	-0.052	-0.583^∗∗^	-0.096	0.143
Visual learning	-0.192	-0.105	0.084	-0.292	-0.156	0.16	-0.059	-0.218	0.2
Reasoning and problem solving	-0.227	-0.04	0.16	0.087	-0.216	0.309	-0.352^∗^	-0.285	0.139
Social cognition	-0.089	-0.168	0.24	-0.026	0.012	0.085	-0.214	-0.335^∗^	0.14
MCCB combine	-0.327^∗^	-0.113	0.21	-0.205	-0.118	0.158	-0.438^∗∗^	-0.341^∗^	0.271

^∗^*FES = First-episode Schizophrenia; CS = Chronic Schizophrenia; HC = Healthy Controls; MMN = Mismatch Negativity; MCCB = the MATRICS consensus cognitive battery; ERSP = event-related spectral perturbation; ^∗^*p* < 0.05; ^∗∗^*p* < 0.01*.

### Evoked Theta Power to the Standard Stimulus

The differences in evoked theta power among the three groups may be found in Table 3. There was also a significant difference among the three groups (*df* = 2, 117, *F* = 10.98, *p* < 0.001). Both patient groups showed significant reductions in the evoked theta power when compared with the HC (*p_*FES*_* = 0.001, *p_*CS*_* < 0.001, Bonferroni-adjusted), yet there was no significant difference between the two patient groups (*p* = 1.000, Bonferroni-adjusted). The Cohen’s effect size for FES was medium (*d* = 0.74), while the Cohen’s effect size for CS was large (*d* = 0.81).

As shown in Table 5, in terms of MCCB domain correlations, all three groups measures were significantly correlated with working memory (*r*_HC_ = 0.361, *p*_HC_ = 0.022, Figure 2B1; *r*_FES_ = 0.347, *p*_FES_ = 0.028, Figure 2B2; *r*_CS_ = 0.408, *p*_CS_ = 0.009, Figure 2B3). However, there were no statistically significant findings on the correlation analysis using the Bonferroni correction, which indicated that the respective correlations were only significant on a descriptive level.

## Discussion

Mismatch negativity generation deficits have been recognized as one of the best potential biomarkers of cognitive impairment in patients with schizophrenia (Näätänen et al., 2016). While MMN deficits have been detected in several deviant types, the most extensively studied are those of duration and frequency, as both of these have been found to be significantly impaired in patients with schizophrenia (Friedman et al., 2012). Some researchers have found differential impairments in frequency and duration MMN and suggested that deficits in duration deviants may be more sensitive indices of MMN reduction during the early stages of schizophrenia (Todd et al., 2008). In our study, compared to HC, frequency MMN was only impaired in CS, but duration MMN was impaired in both FES and CS. Therefore, our results confirmed this viewpoint. A recent meta-analysis (Haigh et al., 2017), which compared the results from several studies that measured MMN reduction in patients with first-episode schizophrenia-spectrum, showed a negligible effect size of 0.04 SD for MMN to frequency deviants and a small-to-medium effect size of 0.47 SD for duration deviants. In the individuals at ultra-high risk (UHR; Koshiyama et al., 2017), duration MMN was significantly smaller when compared with the HC. However, frequency MMN did not differ between the UHR individuals and HC. These similar findings suggest that duration MMN is a better biomarker for the early identification of the patients with schizophrenia than frequency MMN.

Both frequency and duration MMN deficits in CS were more severe when compared with FES, and the within- and between-subject effects were also significant, suggesting that MMN amplitude deficits may correlate with the progression of the patients with schizophrenia. However, MMN deficits were not significantly associated with disease duration in either of the deviant types between the two patient groups, which is in agreement with a recent meta-analysis (Erickson et al., 2016). One conceivable explanation is that MMN impairment worsens within the first 1 to 2 years after the diagnosis, but stabilizes after this critical period. Previously, Salisbury et al. (Salisbury et al., 2007) discovered a progressive course of MMN impairment during the initial 18 months of the disease. Nevertheless, our study did not find that MMN deficits in FES (mean duration of illness was 14.15 months) have any significant relationship with the length of illness. Another possible explanation to this controversy is that MMN deficits may be related to the age of the patients (Todd et al., 2008), yet several studies (Lee et al., 2017b,c), including the current investigation, have shown no correlation between MMN and patient age. Additionally, we did not detect this association with age at illness onset.

While we showed that differential deficits in frequency and duration MMN between FES and CS remain controversial, the correlation study with MCCB showed that the neural processing mechanism of duration and frequency MMN might be different. The results of our study showed that frequency MMN deficits were related to MCCB verbal learning (T-score) in the HC and FES, yet duration MMN deficits were not significantly associated with any cognitive domains in MCCB from either group. This may suggest that frequency MMN is a potential marker that can be correlated with auditory functioning early on in patients with schizophrenia. Similarly, previous researchers found that frequency MMN is intact in FES (Haigh et al., 2017), especially in patients with high premorbid functioning (Salisbury et al., 2017). However, even in these patients, declines in frequency MMN occur over the first few years of the disease in parallel with structural changes in the auditory cortex (Javitt et al., 2000; Kasai et al., 2003). In summary, duration MMN may be related to premorbid aspects of patients with schizophrenia, while frequency MMN may be relevant to the decline in cognitive functioning that occurs during the early stages of patients with schizophrenia (Lee et al., 2017b).

Another important discovery of our study is that deficits in theta power response to standard stimuli were significantly impaired in both groups. Similarly, Lee et al. found that lower frequency oscillations were sparked, especially by the MMN, that may be mapped to the theta frequency range, and that these oscillations are impaired in patients with schizophrenia (Lee et al., 2017a, Lee et al., 2017c). However, their recent study (Lee et al., 2017b) also found that alpha power was significantly impaired in patients with schizophrenia. In return, low-range alpha and theta oscillatory frequencies may contribute to MMN, as they are all impaired in patients with schizophrenia. Our study also found that theta power responses to standard stimuli deficits could not distinguish between FES and CS. More interestingly, theta power response was significantly correlated with MCCB working memory in all three groups. A recent review proposed that theta frequency generation may be tied to the impaired interplay between the cortical pyramidal neurons and local circuit SST-type GABAergic interneurons (Javitt et al., 2017). In addition, another study suggested that working memory may be correlated with GABA levels in patients with schizophrenia (Chen et al., 2014), which may indicate that theta power response to standard stimuli is a marker of auditory working memory in patients with schizophrenia.

## Conclusion

In conclusion, we show the differential MMN measures of deficits between FES and CS. Frequency MMN was not impaired in FES when compared with HC, and it was correlated with MCCB verbal learning. Duration MMN and theta-evoked power were impaired in both patient groups. In addition, duration MMN deficits were not correlated with any MCCB domain, yet theta-evoked power deficits were correlated with MCCB working memory in all three groups. These results suggest that the mechanisms of frequency and duration mismatch negativity and theta power deficits in FES and CS are different, and that the processes may occur during various stages of the disease. Duration MMN may be a more sensitive biomarker during the early stages of patients with schizophrenia, while frequency MMN and theta power response to standard stimuli may be linked to a reduction in the cognitive functioning of patients with schizophrenia.

## Limitations

There are two primary methodological limitation for the current study that should be considered. First, some of the FES patients were already receiving antipsychotic therapy, so we were unable to completely rule out the potential effects of concurrent therapeutic intervention. Secondly, the age at illness onset of CS is generally earlier than that of FES. As we wanted to ensure that age was matched between the subjects, there may be some heterogeneity between the subjects.

## Author Contributions

Y-BX and Q-JB contributed to manuscript preparation. Y-BX and QT performed the neurophysiological data analysis and statistics. Y-BX and YL oversaw MMN data/demographic data collection. C-MW looked over the MMN test. C-YW was in charge of design and implementation of the study and contributed to data interpretation.

## Conflict of Interest Statement

The authors declare that the research was conducted in the absence of any commercial or financial relationships that could be construed as a potential conflict of interest.

## Footnotes

1 http://sccn.ucsd.edu/eeglab/

## References

[B1] Avissar M. Xie S. Vail B. Lopez-Calderon J. Wang Y. Javitt D. C. (2017). Meta-analysis of mismatch negativity to simple versus complex deviants in schizophrenia. *Schizophr. Res.* 191 25–34. 10.1016/j.schres.2017.07.009 28709770 PMC5745291

[B2] Blatow M. Rozov A. Katona I. Hormuzdi S. G. Meyer A. H. Whittington M. A. (2003). A novel network of multipolar bursting interneurons generates theta frequency oscillations in neocortex. *Neuron* 38 805–817. 10.1016/S0896-6273(03)00300-3 12797964

[B3] Catts V. S. Lai Y. L. Weickert C. S. Weickert T. W. Catts S. V. (2016). A quantitative review of the postmortem evidence for decreased cortical N-methyl-D-aspartate receptor expression levels in schizophrenia: how can we link molecular abnormalities to mismatch negativity deficits? *Biol. Psychol.* 116 57–67. 10.1016/j.biopsycho.2015.10.013 26549579

[B4] Chen C. M. A. Stanford A. D. Mao X. Abi-Dargham A. Shungu D. C. Lisanby S. H. (2014). Gaba level, gamma oscillation, and working memory performance in schizophrenia. *NeuroImage Clin.* 4 531–539. 10.1016/j.nicl.2014.03.007 24749063 PMC3989525

[B5] Cohen J. (1988). *Statistical Power Analysis for the Behavioral Sciences*, 2nd Edn. New Jersey, NJ: Lawrence Erlbaum Associates, Inc.

[B6] Elvevåg B. Goldberg T. E. (2000). Cognitive impairment in schizophrenia is the core of the disorder. *Crit. Rev. Neurobiol.* 14 1–21. 10.1615/CritRevNeurobiol.v14.i1.10 11253953

[B7] Erickson M. A. Ruffle A. Gold J. M. (2016). A meta-analysis of mismatch negativity in schizophrenia: from clinical risk to disease specificity and progression. *Biol. Psychiatry* 79 980–987. 10.1016/j.biopsych.2015.08.025 26444073 PMC4775447

[B8] Fisher D. J. Grant B. Smith D. M. Borracci G. Labelle A. Knott V. J. (2011). Effects of auditory hallucinations on the mismatch negativity (MMN) in schizophrenia as measured by a modified ‘optimal’ multi-feature paradigm. *Int. J. Psychophysiol.* 81 245–251. 10.1016/j.ijpsycho.2011.06.018 21749905

[B9] Friedman T. Sehatpour P. Dias E. Perrin M. Javitt D. C. (2012). Differential relationships of mismatch negativity and visual p1 deficits to premorbid characteristics and functional outcome in schizophrenia. *Biol. Psychiatry* 71 521–529. 10.1016/j.biopsych.2011.10.037 22192361 PMC4469217

[B10] Fuentemilla L. Marco-Pallarés J. Münte T. F. Grau C. (2008). Theta EEG oscillatory activity and auditory change detection. *Brain Res.* 1220 93–101. 10.1016/j.brainres.2007.07.079 18076870

[B11] Grzella I. Müller B. W. Oades R. D. Bender S. Schall U. Zerbin D. (2001). Novelty-elicited mismatch negativity in patients with schizophrenia on admission and discharge. *J. Psychiatry Neurosci.* 26 235–246. 11394193 PMC1408303

[B12] Haigh S. M. Coffman B. A. Salisbury D. F. (2017). Mismatch negativity in first-episode schizophrenia: a meta-analysis. *Clin. EEG Neurosci.* 48 3–10. 10.1177/1550059416645980 27170669 PMC5768309

[B13] Hay R. A. Roach B. J. Srihari V. H. Woods S. W. Ford J. M. Mathalon D. H. (2015). Equivalent mismatch negativity deficits across deviant types in early illness schizophrenia-spectrum patients. *Biol. Psychol.* 105 130–137. 10.1016/j.biopsycho.2015.01.004 25603283 PMC4336819

[B14] He Y. L. Zhang M. Y. (1997). The positive and negative syndrome scale (PANSS) and its application. *J. Clin. Psychiatry* 7 353–355.

[B15] Hermens D. F. Ward P. B. Hodge M. A. R. Kaur M. Naismith S. L. Hickie I. B. (2010). Impaired MMN/p3a complex in first-episode psychosis: cognitive and psychosocial associations. *Prog. Neuropsychopharmacol. Biol. Psychiatry* 34 822–829. 10.1016/j.pnpbp.2010.03.019 20302901

[B16] Higuchi Y. Sumiyoshi T. Seo T. Miyanishi T. Kawasaki Y. Suzuki M. (2013). Mismatch negativity and cognitive performance for the prediction of psychosis in subjects with at-risk mental state. *PLoS One* 8:e54080. 10.1371/journal.pone.0054080 23349791 PMC3547932

[B17] Higuchi Y. Tomonori S. Tomohiro M. Yasuhiro K. Michio S. Tomiki S. (2014). Mismatch negativity and p3a/reorienting complex in subjects with schizophrenia or at-risk mental state. *Front. Behav. Neurosci.* 8:172. 10.3389/fnbeh.2014.00172 24860454 PMC4026722

[B18] Hsiao F. J. Wu Z. A. Ho L. T. Lin Y. Y. (2009). Theta oscillation during auditory change detection: an MEG study. *Biol. Psychol.* 81 58–66. 10.1016/j.biopsycho.2009.01.007 19428969

[B19] Javitt D. C. (2015). Neurophysiological models for new treatment development in schizophrenia: early sensory approaches. *Ann. N. Y. Acad. Sci.* 1344 92–104. 10.1111/nyas.12689 25721890 PMC4467888

[B20] Javitt D. C. Freedman R. (2015). Sensory processing dysfunction in the personal experience and neuronal machinery of schizophrenia. *Am. J. Psychiatry* 172 17–31. 10.1176/appi.ajp.2014.13121691 25553496 PMC4501403

[B21] Javitt D. C. Lee M. Kantrowitz J. T. Martinez A. (2017). Mismatch negativity as a biomarker of theta band oscillatory dysfunction in schizophrenia. *Schizophr. Res.* 191 51–60. 10.1016/j.schres.2017.06.023 28666633

[B22] Javitt D. C. Shelley A. M. Silipo G. Lieberman J. A. (2000). Deficits in auditory and visual context-dependent processing in schizophrenia. *Arch. Gen. Psychiatry* 57 1131–1137. 10.1001/archpsyc.57.12.1131 11115326

[B23] Javitt D. C. Sweet R. A. (2015). Auditory dysfunction in schizophrenia: integrating clinical and basic features. *Nat. Rev. Neurosci.* 16 535–550. 10.1038/nrn4002 26289573 PMC4692466

[B24] Kantrowitz J. T. Epstein M. L. Beggel O. Rohrig S. Lehrfeld J. M. Revheim N. (2016). Neurophysiological mechanisms of cortical plasticity impairments in schizophrenia and modulation by the nmda receptor agonist d-serine. *Brain* 139 3281–3295. 10.1093/brain/aww262 27913408 PMC5840885

[B25] Kantrowitz J. T. Epstein M. L. Lee M. Lehrfeld N. Nolan K. A. Shope C. (2017). Improvement in mismatch negativity generation during, d -serine treatment in schizophrenia: correlation with symptoms. *Schizophr. Res.* 191 70–79. 10.1016/j.schres.2017.02.027 28318835

[B26] Kasai K. Shenton M. E. Salisbury D. F. Hirayasu Y. Mccarley R. W. (2003). Progressive decrease of left heschl gyrus and planum temporale gray matter volume in first-episode schizophrenia. *Arch. Gen. Psychiatry* 60 766–775. 10.1001/archpsyc.60.8.766 12912760 PMC2901861

[B27] Koshiyama D. Kirihara K. Tada M. Nagai T. Koike S. Suga M. (2017). Duration and frequency mismatch negativity shows no progressive reduction in early stages of psychosis. *Schizophr. Res.* 190 32–38. 10.1016/j.schres.2017.03.015 28314681

[B28] Lee M. Balla A. Sershen H. Sehatpour P. Lakatos P. Javitt D. C. (2017a). Rodent mismatch negativity (mmn)/theta neuro-oscillatory response as a translational neurophysiological biomarker for n-methyl-d-aspartate receptor-based new treatment development in schizophrenia. *Neuropsychopharmacology* 43 571–582. 10.1038/npp.2017.176 28816240 PMC5770758

[B29] Lee M. Sehatpour P. Dias E. C. Silipo G. S. Kantrowitz J. T. Martinez A. M. (2017b). A tale of two sites: differential impairment of frequency and duration mismatch negativity across a primarily inpatient versus a primarily outpatient site in schizophrenia. *Schizophr. Res.* 191 10–17. 10.1016/j.schres.2017.07.032 28779851

[B30] Lee M. Sehatpour P. Hoptman M. J. Lakatos P. Dias E. C. Kantrowitz J. T. (2017c). Neural mechanisms of mismatch negativity dysfunction in schizophrenia. *Mol. Psychiatry* 108 37–37. 10.1038/mp.2017.3 28167837 PMC5547016

[B31] Magno E. Yeap S. Thakore J. H. Garavan H. Sanctis P. D. Javitt D. C. (2008). Are auditory-evoked frequency and duration mismatch negativity (MMN) deficits endophenotypic for schizophrenia? high-density electrical mapping in clinically unaffected first-degree relatives, first-episode and chronic schizophrenia. *Biol. Psychiatry* 64 385–391. 10.1016/j.biopsych.2008.03.019 18472090 PMC3057136

[B32] Makeig S. Jung T. P. Bell A. J. Sejnowski G. T. J. (1997). Blind separation of auditory event-related brain responses into independent components. *Proc. Natl. Acad. Sci. U.S.A.* 94 10979–10984. 10.1073/pnas.94.20.10979 9380745 PMC23551

[B33] Mondragón-Maya A. Solís-Vivanco R. León-Ortiz P. Rodríguez-Agudelo Y. Yáñez-Téllez G. Bernal-Hernández J. (2013). Reduced p3a amplitudes in antipsychotic naïve first-episode psychosis patients and individuals at clinical high-risk for psychosis. *J. Psychiatr. Res.* 47 755–761. 10.1016/j.jpsychires.2012.12.017 23507048

[B34] Näätänen R. Sussman E. S. Salisbury D. Shafer V. L. (2014). Mismatch negativity (MMN) as an index of cognitive dysfunction. *Brain Topogr.* 27 451–466. 10.1007/s10548-014-0374-6 24838819 PMC4096841

[B35] Näätänen R. Tervaniemi M. Sussman E. Paavilainen P. Winkler I. (2001). ‘primitive intelligence’ in the auditory cortex. *Trends Neurosci.* 24 283–288. 10.1016/S0166-2236(00)01790-2 11311381

[B36] Näätänen R. Todd J. Schall U. (2016). Mismatch negativity (MMN) as biomarker predicting psychosis in clinically at-risk individuals. *Biol. Psychol.* 116 36–40. 10.1016/j.biopsycho.2015.10.010 26542526

[B37] Oranje B. Aggernaes B. Rasmussen H. Ebdrup B. H. Glenthøj B. Y. (2017). Selective attention and mismatch negativity in antipsychotic- naïve, first-episode schizophrenia patients before and after 6 months of antipsychotic monotherapy. *Psychol. Med.* 47 2155–2165. 10.1017/S0033291717000599 28443529

[B38] Pang Y. X. Zhang J. Yang C. L. Cang Y. Wang X. L. (2011). Application of WAIS-RC short forms and adult intelligence disability scale in mental impairment assessment. *Fa Yi Xue Za Zhi* 27 189–192. 21899009

[B39] Perez V. B. Woods S. W. Roach B. J. Ford J. M. Mcglashan T. H. Srihari V. H. (2014). Automatic auditory processing deficits in schizophrenia and clinical high-risk patients: forecasting psychosis risk with mismatch negativity. *Biol. Psychiatry* 75 459–469. 10.1016/j.biopsych.2013.07.038 24050720 PMC4028131

[B40] Randeniya R. Oestreich L. K. L. Garrido M. I. (2018). Sensory prediction errors in the continuum of psychosis. *Schizophr. Res.* 191 109–122. 10.1016/j.schres.2017.04.019 28457774

[B41] Salisbury D. F. Kuroki N. Kasai K. Shenton M. E. Mccarley R. W. (2007). Progressive and interrelated functional and structural evidence of post-onset brain reduction in schizophrenia. *Arch. Gen. Psychiatry* 64 521–529. 10.1001/archpsyc.64.5.521 17485604 PMC2903200

[B42] Salisbury D. F. Polizzotto N. R. Nestor P. G. Haigh S. M. Koehler J. Mccarley R. W. (2017). Pitch and duration mismatch negativity and premorbid intellect in the first hospitalized schizophrenia spectrum. *Schizophr. Bull.* 43 407–416. 10.1093/schbul/sbw074 27231308 PMC5605266

[B43] Salisbury D. F. Shenton M. E. Griggs C. B. Bonner-Jackson A. McCarley R. W. (2002). Mismatch negativity in chronic schizophrenia and first-episode schizophrenia. *Arch. Gen. Psychiatry* 59 686–694. 10.1001/archpsyc.59.8.686 12150644

[B44] Shi C. Kang L. Yao S. Ma Y. Li T. Liang Y. (2015). The MATRICS consensus cognitive battery (MCCB): co-norming and standardization in china. *Schizophr. Res.* 169 109–115. 10.1016/j.schres.2015.09.003 26441005 PMC4916953

[B45] Thönnessen H. Zvyagintsev M. Harke K. C. Boers F. Dammers J. Mathiak K. (2008). Optimized mismatch negativity reflects deficits in schizophrenia patients in a combined EEG and MEG study. *Eur. Psychiatry* 23 S135–S135. 10.1016/j.eurpsy.2008.01.852 18060677

[B46] Todd J. Michie P. T. Schall U. Karayanidis F. Yabe H. Näätänen R. (2008). Deviant matters: duration, frequency, and intensity deviants reveal different patterns of mismatch negativity reduction in early and late schizophrenia. *Biol. Psychiatry* 63 58–64. 10.1016/j.biopsych.2007.02.016 17585889

[B47] Toyomaki A. Kusumi I. Matsuyama T. Kako Y. Ito K. Koyama T. (2008). Tone duration mismatch negativity deficits predict impairment of executive function in schizophrenia. *Prog. Neuropsychopharmacol. Biol. Psychiatry* 32 95–99. 17764800 10.1016/j.pnpbp.2007.07.020

[B48] Umbricht D. Javitt D. Novak G. Bates J. Pollack S. Lieberman J. (1999). Effects of risperidone on auditory event-related potentials in schizophrenia. *Int. J. Neuropsychopharmacol.* 2 299–304. 10.1017/S1461145799001595 11285146

[B49] Umbricht D. Krljes S. (2005). Mismatch negativity in schizophrenia: a meta-analysis. *Schizophr. Res.* 76 1–23. 10.1016/j.schres.2004.12.002 15927795

[B50] Weickert T. W. Goldberg T. E. Gold J. M. Bigelow L. B. Egan M. F. Weinberger D. R. (2000). Cognitive impairments in patients with schizophrenia displaying preserved and compromised intellect. *Arch. Gen. Psychiatry* 57 907–913. 10.1001/archpsyc.57.9.907 10986554

[B51] Womelsdorf T. Valiante T. A. Sahin N. T. Miller K. J. Tiesinga P. (2014). Dynamic circuit motifs underlying rhythmic gain control, gating and integration. *Nat. Neurosci.* 17 1031–1039. 10.1038/nn.3764 25065440

[B52] Wynn J. K. Sugar C. Horan W. P. Kern R. Green M. F. (2010). Mismatch negativity, social cognition, and functioning in schizophrenia patients. *Biol. Psychiatry* 67 940–947. 10.1016/j.biopsych.2009.11.024 20074704 PMC2862843

